# Vitamin E prevents steroid-induced osteonecrosis in rabbits

**DOI:** 10.3109/17453671003628772

**Published:** 2010-03-31

**Authors:** Masaaki Kuribayashi, Mikihiro Fujioka, Kenji A Takahashi, Yuji Arai, Masashi Ishida, Tsuyoshi Goto, Toshikazu Kubo

**Affiliations:** Department of Orthopaedics, Graduate School of Medical Science, Kyoto Prefectural University of Medicine, KyotoJapan

## Abstract

**Background and purpose:**

Prevention of osteonecrosis after corticosteroid administration would be important. We examined the potential of vitamin E (**α**-tocopherol) to reduce the incidence of corticosteroid-induced osteonecrosis in an animal model.

**Methods:**

Japanese white rabbits were divided into 2 groups; the control group was fed a normal diet and the experimental group was fed **α**-tocopherol-supplemented diet in which **α**-tocopherol (600 mg/kg diet) was added to the normal diet. To induce osteonecrosis, high-dose methylprednisolone acetate (MPSL) (20 mg/kg body weight) was injected once into the right gluteus medius muscle of all rabbits. 4 weeks after the injection of MPSL, the presence or absence of osteonecrosis of bilateral femurs was examined histopathologically. The tocopherol/cholesterol ratios were calculated. The plasma levels of thiobarbituric acid-reactive substances (TBARS) were measured.

**Results:**

Alpha-tocopherol-supplemented diet reduced the incidence of osteonecrosis, which developed in 14 of 20 rabbits in the control group and 5 of 21 rabbits in the experimental group (p = 0.004). The tocopherol/cholesterol ratio was higher in the experimental group than in the control group after the **α**-tocopherol administration. The plasma TBARS level was lower in the experimental group than in the control group at 4 weeks after the MPSL administration.

**Interpretation:**

Our findings may offer a new approach for the prevention of corticosteroid-induced osteonecrosis.

## Introduction

Osteonecrosis of the hip occurs more frequently in patients who receive high-dose corticosteroid therapy for the treatment of diseases including systemic lupus erythematosus and inflammatory bowel disease, and also for immunosuppression after renal transplants (Spencer and Maisey 1985, Zizic et al. 1985, Vakil and Sparberg 1989). When osteonecrosis involves a large volume of the femoral head, it collapses in many cases with a need for arthroplasty. Since corticosteroid-induced osteonecrosis of the hip tends to occur in relatively young patients, there is concern for the durability of prostheses with long-term use (Brinker et al. 1994, Chiu et al. 1997).

There is consensus that one of the causal factors of osteonecrosis is the ischemic status inside the bone that forms after the administration of corticosteroid (Drescher et al. 2004). Various mechanisms have been proposed regarding the way in which corticosteroids induce osteonecrosis, for example hyperlipidemia, fat emboli, hypercoagulable condition, vascular endothelial dysfunction, and apoptosis of bone tissues (Wang et al. 1977, Jones 1985, 1992, Kabata et al. 2000, Iuchi et al. 2003, Drescher et al. 2004). Even so, previous studies have been unable to clarify the detailed mechanism of the process by which corticosteroid administration induces the development of osteonecrosis. Recently, it was reported that using a rabbit model, oxidative injury was present in the bone shortly after corticosteroid administration and before the development of osteonecrosis (Ichiseki et al. 2005). Accordingly, we believe that antioxidative substances may alleviate oxidative injury following corticosteroid administration, and thus prevent osteonecrosis. Vitamin E is a fat-soluble substance in the body that has potent antioxidant properties (Burton et al. 1983). Various homologs of vitamin E exist in nature, and 8 substances including 4 types of tocopherols and 4 types of tocotrienols have been identified. The biopotencies of tocopherols (relative to that of α-tocopherol, at 100) are 30–50 for β-tocopherol, 10 for γ-tocopherol, and 2 or less for δ-tocopherol (Weiser and Vecchi 1982). The biopotencies of tocotrienols are known to be lower than tocopherols (Weiser and Vecchi 1982). Thus α-tocopherol, which has the highest potency of the known vitamin E homologs, should have the greatest potential for the prevention of osteonecrosis.

In this study, we examined the capacity of α-tocopherol to reduce the incidence of corticosteroid-induced osteonecrosis in an animal model. We also examined whether α-tocopherol could reduce the number of sites of osteonecrosis development in individual rabbits that developed osteonecrosis. Finally, we determined whether α-tocopherol exerts some effects on disorders of lipid metabolism, lipid peroxidation, and vascular damage after corticosteroid administration.

## Material and methods

### Animals and diets

All protocols in this study were followed in accordance with the guidelines of the Animal Care and Use Committee of our institution (date of issue: March 31, 2006; registration number: 16-23).

Male Japanese white rabbits aged 28–32 weeks (Kitayama Labs Co. Ltd., Nagano, Japan) were kept in separate cages under controlled photoperiodic conditions comprising 12 h of light and 12 h of darkness at a temperature of 24 ± 1 °C. Their body weight was recorded on a weekly basis (baseline weight: 3.3–3.9 kg). Water was provided by an automatic watering system. 50 rabbits were divided into two groups. Rabbits in the control group (n = 25) were fed with commercially available normal diet (ORC4; Oriental Yeast Co. Ltd., Tokyo, Japan), while those in the experimental group (n = 25) were fed with ORC4 diet supplemented with α-tocopherol, which contained 600 mg α-tocopherol per kg. The ORC4 diet normally contains 0.71 mg α-tocopherol per kg of diet. Alpha-tocopherol-supplemented diet was prepared by the manufacturer (Oriental Yeast) by adding α-tocopherol at the ratio of 600 mg/kg diet to the ingredients of ORC4 during the manufacturing process.

### A rabbit model of osteonecrosis

We used a rabbit model of corticosteroid-induced osteonecrosis (Yamamoto et al. 1997). 2 weeks after the start of feeding and after allocation of the rabbits into the 2 groups, a high dose of methylprednisolone acetate (MPSL) (20 mg/kg body weight; Pfizer Japan Inc., Tokyo, Japan) was administered once into the right gluteus medius muscle of all the rabbits to induce osteonecrosis.

### Tissue preparation

4 weeks after administration of MPSL, the rabbits were killed with an excessive dose of intravenously injected sodium pentobarbital. The bilateral femurs were immediately harvested and divided at the distal one-third and proximal one-third. The femurs were fixed in 10% buffered formalin for 1 week and then decalcified with 10% EDTA. After decalcification, the femurs were embedded in paraffin. 4-μm-thick coronal sections of the femurs were prepared and stained with hematoxylin and eosin.

### Immunohistochemistry

To examine the development of lipid peroxidation and vascular damage in bone, the femurs were stained immunohistochemically with anti-malondialdehyde monoclonal antibody (clone 1F83) (Yamada et al. 2001). Briefly, after deparaffinization, the sections were treated with 3% H_2_O_2_ for 10 min, rinsed in PBS (pH 7.6), and pretreated with 3% non-immune animal serum in PBS for 30 min at room temperature. Then the sections were reacted with monoclonal antibody to malondialdehyde (clone 1F83) overnight at 4°C. Sections were stained by the avidin-biotin complex method. Sections were then treated with DAB, and counterstaining was carried out with hematoxylin.

### Evaluation of osteonecrosis

The presence or absence of osteonecrosis was determined in 4 sections taken from the distal and proximal ends of both femurs of each rabbit. The sections were examined histopathologically by two researchers who were kept blind regarding the identity of the treatment group. When the two examiners did not agree, they discussed the findings (still blind) to reach a consensus. A positive diagnosis of osteonecrosis was determined based on the diffuse presence of empty lacunae or pyknotic nuclei of osteocytes within the bone trabeculae, accompanied by surrounding bone marrow cell necrosis or fat cell necrosis (Yamamoto et al. 1997). Empty lacunae of osteocytes within the bone trabeculae without bone marrow cell necrosis or fat cell necrosis was not diagnosed as osteonecrosis. If osteonecrosis was found in at least 1 of the 4 sections, the rabbit was considered to have osteonecrosis. The number of rabbits that developed osteonecrosis, the source of the bone tissue, and the number of osteonecrosis sites that developed in each rabbit were recorded.

### Measurement of the size and area of fat cells in bone marrow

The effect of α-tocopherol on corticosteroid-induced adipogenesis was assessed by comparing the mean bone marrow fat cell size and the mean bone marrow fat cell area in the control group and in the experimental group. The effect of adipogenesis on osteonecrosis was also assessed by comparing the mean fat cell size and the mean fat cell area in the rabbits that developed osteonecrosis (the ON+ group) and in the rabbits that did not develop osteonecrosis (the ON- group). The bone marrow fat cell size was calculated as an average of the greatest diameters of 100 fat cells in 4 randomly-selected non-necrotic fields (one field = 25 × 10^-8^ m^2^) from the proximal one-third of the femur using image analysis software (ImageJ version 1.41o; National Institutes of Health, MD) (Parfitt et al. 1987, Pengde et al. 2008). The fat cell area, including the necrotic area of the marrow fat cells, and the trabecular bone within the femoral head proximal to a straight line connecting the edges of the articular cartilage (6 sections from each rabbit) was quantitated histomorphometrically with the Mocha program based on the method suggested by the American Society of Bone and Mineral Research Histomorphometry Nomenclature Committee (Parfitt et al. 1987, Pengde et al. 2008).

### Evaluation of lipid peroxidation and vascular damage in bone

The effect of α-tocopherol on corticosteroid-induced lipid peroxidation and vascular damage was assessed by comparing immunohistological data in the control group and in the experimental group. The effect of corticosteroid-induced lipid peroxidation and vascular damage on osteonecrosis was also assessed by comparing immunohistological data between the ON+ group and the ON- group. The anti-malondialdehyde antibody-positive (MDA+) marrow vessels were counted in 4 randomly-selected fields from the proximal one-third of the femur (one field = 25 × 10^-8^ m^2^).

### Estimation of *α*-tocopherol intake

The food intake of a rabbit was calculated by subtracting the weight of the remaining food in the hopper from the weight of the food supplied. The intake of α-tocopherol (mg/kg/day) was calculated based on these values.

### Hematological examination

Blood samples were collected in the morning, from the auricular artery of each rabbit in a fasting state. Samples were collected at the start of the study (week –2), immediately before the administration of MPSL (week 0), 2 weeks after the administration of MPSL (week 2), and 4 weeks after the administration (week 4). Heparin was added to the blood samples and they were centrifuged at 3,000 rpm for 15 min at 4°C. The supernatant was stored under light-shielded conditions at –80°C. The plasma levels of tocopherol (mg/dL) were measured by the fluorescence method (Thompson et al. 1973) (Mitsubishi Chemical Medience Corporation, Tokyo, Japan). The plasma levels of total cholesterol (T-cho; mg/dL) and triglycerides (TG; mg/dL) were measured using an automated analyzer. The extent of lipid peroxidation was determined as thiobarbituric acid-reactive substances (TBARS) in plasma. The plasma levels of TBARS (μM) were measured using the Malondialdehyde Assay kit (NWK-MD01; Northwest, Vancouver, WA). The measurement principle of TBARS is as follows: malondialdehyde (MDA) in plasma reacts with thiobarbituric acid (TBA) and forms MDA-TBA_2_, which has strong absorbance at a wavelength of 532 nm. The level of MDA-TBA_2_ is quantified by spectrophotometry. Plasma tocopherol concentrations are known to be dependent on total cholesterol concentration (Vatassery et al. 1983). Thus, the tocopherol/cholesterol ratios were calculated.

### Statistics

Categorical data, i.e. incidence of osteonecrosis, was analyzed using Fisher's exact probability test. Numerical data in each group were expressed as means and standard deviation (SDs). Simple comparisons of numerical data were performed using Student's t-test. Longitudinal numerical data, i.e. hematological data, at 4 time points (week –2, week 0, week 2, and week 4) were expressed as means and SDs and were analyzed using the Tukey-Kramer method for all pairwise comparisons with α set a priori to 0.05. Statistical analyses were performed using Excel 2007 (Microsoft) with the add-on software Statcel 2 (Yanai H, 2004) and with p-values of < 0.05 considered statistically significant.

## Results

### Incidence of osteonecrosis

**Figure 1.**
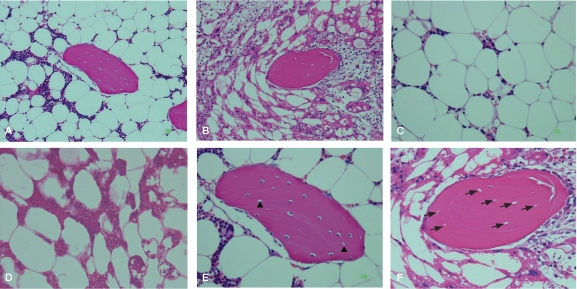
Histological features of osteonecrosis in rabbits. A. Nomal bone harvested from a comparable rabbit not treated with α-tocopherol nor treated with methylprednisolone acetate. B. Typical osteonecrotic lesion (experimental group). C. Nomal bone marrow cells. D. Necrotic bone marrow cells. Bone marrow cells had necrosis and stained acidophilic. The nuclei of bone marrow cells displayed pyknosis and karyorrhexis. The cellular structure of fat cells collapsed. E. Normal bone. Empty lacunae of the osteocytes (arrowhead) without bone marrow cell necrosis or fat cell necrosis. F. Osteonecrotic bone. Bone cells in the bone trabeculae showed pyknosis and empty lacunae (arrow) that were associated with necrotic changes of the surrounding bone marrow cells. Stain: hematoxylin and eosin; magnification: ×200 (A, B), ×400 (C, D, E, F).

9 of the 50 rabbits died after the administration of MPSL and were excluded from the evaluation. 5 were in the control group and 4 were in the experimental group. 14 of the 20 rabbits in the control group and 5 of the 21 rabbits in the experimental group developed osteonecrosis (Figure 1). The α-tocopherol-supplemented diet reduced the incidence of osteonecrosis (p = 0.004, Fisher's exact probability test). There was no difference in the number of osteonecrosis sites in each rabbit that developed osteonecrosis between the two groups (control group: 1.57 (SD 0.65); experimental group: 1.80 (SD 0.45); p = 0.5).

### Size and area of bone marrow fat cells

The diameter of fat cells was 54 (SD 7.0) μm in the control group and 56 (SD 3.7) μm in the experimental group (p = 0.3). The diameter of fat cells was 54 (SD 4.3) μm in the ON+ group and 55 (SD 6.7) μm in the ON- group (p = 0.5). The area of fat cells was 57% (SD 6.6) in the control group, and 57% (SD 6.8) in the experimental group (p = 0.9). The area of fat cells was 57% (SD 6.5) in the ON+ group and 56% (SD 7.0) in the ON- group (p = 0.7).

### Lipid peroxidation and vascular damage in bone

**Figure 2.**
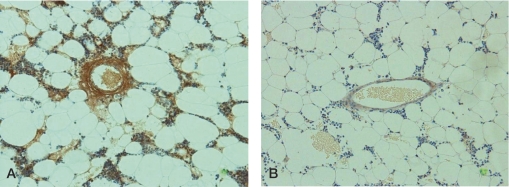
A. Strong immunoreactivity found in both a marrow vessel and bone marrow cells. B. Weak immunoreactivity found in both a marrow vessel and bone marrow cells. Stain: anti-malondialdehyde monoclonal antibody (clone 1F83); magnification: ×200.

By immunostaining with anti-malondialdehyde antibody (clone 1F83), vessels in the bone marrow and bone marrow cells were stained brown (Figure 2). The number of MDA+ vessels was 3.9 (SD 2.3) in the control group, and 1.4 (SD 0.96) in the experimental group (p < 0.001). The MDA+ vessel counts were 3.9 (SD 2.1) in the ON+ group, and 1.5 (SD 1.4) in the ON- group (p = 0.001).

### Intake of α-tocopherol

The intake of food during the experimental period was 4,987 (SD 542) g in the control group, and 4,981 (SD 541) g in the experimental group (NS). The intake of α-tocopherol was 0.02 (SD 0.01) mg/kg/day in the control group and 20.3 (SD 5.9) mg/kg/day in the experimental group (p < 0.001).

### Hematological findings

**Figure 3.**
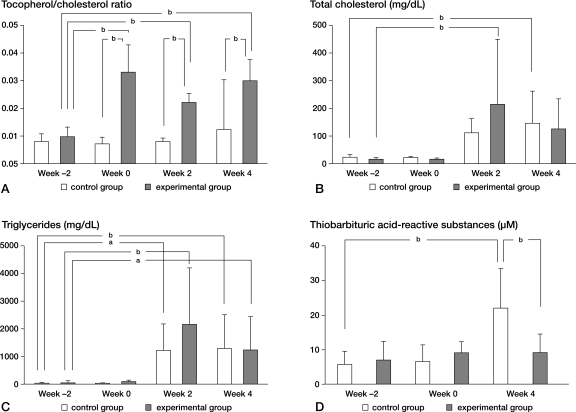
Hematological findings. The tocopherol/cholesterol ratios (A), total cholesterol levels (B), triglyceride levels (C), and concentration of thiobarbituric acid-reactive substances (D) are shown. **^a^** P < 0.05, **^b^** P < 0.01 by Tukey-Kramer method for between-group and within-group comparisons).

Hematological data for the control group and the experimental group were compared at the same time points (between-group comparison), and between week 2 (baseline) and the 3 other time points in the group (within-group comparison) (Figure 3). The tocopherol/cholesterol ratios in the experimental group increased during the period from week 0 to week 4 (p < 0.01). Regarding plasma levels of total cholesterol (T-cho) and triglycerides (TG), there was no difference between the groups at the same time points. The plasma T-cho level in the experimental group at week 2 and that in the control group at week 4 were higher than the baseline level (p < 0.01). The plasma levels of TG increased during the period from week 2 to week 4 in both groups (p < 0.01). Hemolysis and lipemia were considered to lead to measurement errors of plasma TBARS levels for the following reasons. Hemoglobin, which is released by hemolysis (the collapse of red blood cells) has an absorbance peak around the same wavelength as that of MDA-TBA_2_, which affects the TBARS value measured by spectrophotometry. Also, lipemia, which means high levels of lipids in plasma, affects measurement values by spectrophotometry by raising background levels. Thus, we excluded samples with high levels of hemolysis and lipemia, considering that we could not overlook the potential error caused by the effect of these on TBARS level—the index of lipid peroxidation for which precision is especially required in our study design. All samples obtained 2 weeks after the MPSL administration (except 1 plasma sample in the control group) were severely lipemic and were thus excluded from the statistical analysis. The plasma TBARS level at week 4 in the experimental group was lower than that in the control group (p < 0.01).

## Discussion

Alpha-tocopherol statistically significantly reduced the incidence of osteonecrosis in this rabbit model of corticosteroid-induced osteonecrosis. This finding suggests that the intake of α-tocopherol may prevent corticosteroid-induced osteonecrosis. However, there were still rabbits that developed osteonecrosis. Alpha-tocopherol did not reduce the number of sites of osteonecrosis development in individual rabbits that developed osteonecrosis. We found no influence of α-tocopherol on the severity of osteonecrosis. The results indicate that the cause of osteonecrosis is multifactorial (Wang et al. 1977, Jones 1985, 1992, Kabata et al. 2000, Iuchi et al. 2003, Drescher et al. 2004, Zhang et al. 2009). There may be a threshold in the development of osteonecrosis, which, once reached, may mean the initiation of development of osteonecrosis even though prophylactic treatment is given.

We evaluated disorders of lipid metabolism by measuring T-cho and TG with a biochemical approach, as well as fat cell size and area by histology. T-cho and TG levels increased after the administration of MPSL to a similar extent in the experimental group and the control group. Regarding the size and area of fat cells, no statistically significant differences were found between the experimental group and the control group and between the ON+ and ON- groups. Other researchers reported that disorders of lipid metabolism was osteonecrosis-prevention mechanism (Motomura et al. 2004, Pengde et al. 2008, Zhang et al. 2009). Disorders of lipid metabolism was not improved by α-tocopherol. These results indicate that some other osteonecrosis-prevention mechanisms exist other than disorders of lipid metabolism.

We also evaluated vascular damage caused by oxidative stress by measuring TBARS with a biochemical approach and by counting the number of MDA+ vessels using a histological approach. The TBARS level and the number of MDA+ vessels were lower in the experimental group than in the control group. Alpha-tocopherol reduced oxidative stress in the blood and in the blood vessels. Vascular endothelial cells that are constantly exposed to blood flow are susceptible to injury by oxidative stress (Schilling and Elliott 1992). When they are subjected to oxidative stress, the production of nitric monoxide (NO), which is an endothelium-derived vascular relaxation factor, becomes reduced (Niu et al. 1994). Since NO has an anti-platelet effect through elevation of cyclic GMP levels in the platelets, oxidative stress suppresses this anti-platelet effect from the reduction of NO production. Also, vascular endothelial damage due to oxidative stress reduces thrombomodulin production. When the coagulation system is activated locally, thrombin is produced but immediately inactivated by binding with thrombomodulin on vascular endothelial cells. Also, the complex of thrombin and thrombomodulin activates the protein C-dependent anticoagulant pathway. In short, vascular endothelial damage by oxidative stress the anticoagulation system through reduced thrombomodulin production (Lentz et al. 1999). Thus, we believe that α-tocopherol suppressed vascular damage by reducing oxidative stress in the blood and blood vessels, thereby maintaining anti-platelet and anticoagulant effects. We infer that this is one of the mechanisms by which α-tocopherol prevents the development of steroid-induced osteonecrosis.

Various hypotheses have been put forward for the etiological factors involved in corticosteroid-induced osteonecrosis, including disorders of lipid metabolism and abnormalities in the coagulation and fibrinolytic system (Wang et al. 1977, Jones 1985, 1992, Drescher et al. 2004). Based on these hypotheses, several animal studies have been done in an attempt to establish methods to prevent corticosteroid-induced osteonecrosis. It has been reported that single or combined administration of probucol (a lipid-lowering agent) and/or warfarin (an anticoagulant) is effective in the prevention of osteonecrosis in rabbits (Motomura et al. 2004). It has also been reported that pravastatin and simvastatin treatments have a preventative effect against osteonecrosis in rabbits but that this is not the case with probucol treatment, and that all three agents suppress elevation of lipid levels (Iwakiri et al. 2008). Also, icaritin has been reported to exert a dose-dependent effect on reducing the incidence of osteonecrosis with inhibition of intravascular thrombosis and extravascular lipid deposition (Zhang et al. 2009). From these reports, it is likely that there exist multiple mechanisms for the suppression of osteonecrosis in addition to the reduction of lipid levels (Motomura et al. 2004, Iwakiri et al. 2008, Zhang et al. 2009). Other pathophysiological events may also play an important role in the development of osteonecrosis, such as the corticosteroid-induced hypercoagulable condition and genetic predisposition, which can lead to intravascular coagulation and microcirculatory disturbance (Zhang et al. 2009). Our hypothesis is supported by the report by Zhang et al.

Many biochemical and biological studies have been done using α-tocopherol, and its antioxidant, membrane stabilizing, and microcirculation activation effects have been reported (Burton et al. 1983, Tasinato et al. 1995). Our hypothesis is supported by the results of one study in which oxidative injury occurred after corticosteroid administration but before the development of osteonecrosis, and by another study showing that glutathione, which is also an antioxidant, suppressed osteonecrosis (Ichiseki et al. 2004, 2005).

It has been reported that α-tocopherol is a safe agent and that its upper limit of no observed adverse effect level (NOAEL) in humans when used as a medicine is 1,600 mg/day (Kappus and Diplock 1992). The amount of α-tocopherol that each rabbit consumed in this study was equivalent to about 1,2 mg/day for a man (60 kg).

Our study has the following limitations. Because the occurrence of osteonecrosis was evaluated at only one time point, 4 weeks after the MPSL administration, it is possible that osteonecrosis had been repaired before this time point or that the development of osteonecrosis was delayed. Because we limited the intake of α-tocopherol to 600 mg/kg diet, we were unable to determine the optimum level of α-tocopherol that can suppress the development of osteonecrosis. Thus, future studies should determine the timing of osteonecrosis development and the level of α-tocopherol required to elicit this suppressive effect on the development.

In this study, α-tocopherol was administered orally to rabbits (in their food) at a safe dose and it suppressed corticosteroid-induced osteonecrosis. Since α-tocopherol is safe and is frequently used in clinical settings, we believe that this agent could easily be tested in clinical trials to confirm its effect on prevention of osteonecrosis in humans, although further studies are required before that.
